# TBAJ-876 Retains Bedaquiline’s Activity against Subunits c and ε of *Mycobacterium tuberculosis* F-ATP Synthase

**DOI:** 10.1128/AAC.01191-19

**Published:** 2019-09-23

**Authors:** Jickky Palmae Sarathy, Priya Ragunathan, Joon Shin, Christopher B. Cooper, Anna M. Upton, Gerhard Grüber, Thomas Dick

**Affiliations:** a Department of Medicine, Yong Loo Lin School of Medicine, National University of Singapore, Singapore; b School of Biological Sciences, Nanyang Technological University, Singapore; c Global Alliance for TB Drug Development (TB Alliance), New York, New York, USA; d Department of Microbiology and Immunology, Yong Loo Lin School of Medicine, National University of Singapore, Singapore; e Center for Discovery and Innovation, Hackensack Meridian Health, Nutley, New Jersey, USA

**Keywords:** ε subunit, F-ATP synthase, TBAJ-876, bedaquiline, c subunit, diarylquinoline

## Abstract

The antituberculosis drug bedaquiline (BDQ) inhibits Mycobacterium tuberculosis F-ATP synthase by interfering with two subunits. Drug binding to the c subunit stalls the rotation of the c ring, while binding to the ε subunit blocks coupling of c ring rotation to ATP synthesis at the catalytic α_3_:β_3_ headpiece. BDQ is used for the treatment of drug-resistant tuberculosis. However, the drug is highly lipophilic, displays a long terminal half-life, and has a cardiotoxicity liability by causing QT interval prolongation. Recent medicinal chemistry campaigns have resulted in the discovery of 3,5-dialkoxypyridine analogues of BDQ that are less lipophilic, have higher clearance, and display lower cardiotoxic potential. TBAJ-876, which is a new developmental compound of this series, shows attractive antitubercular activity and efficacy in a murine tuberculosis model. Here, we asked whether TBAJ-876 and selected analogues of the compound retain BDQ’s mechanism of action. Biochemical assays showed that TBAJ-876 is a potent inhibitor of mycobacterial F-ATP synthase. Selection of spontaneous TBAJ-876-resistant mutants identified missense mutations at BDQ’s binding site on the c subunit, suggesting that TBAJ-876 retains BDQ’s targeting of the c ring. Susceptibility testing against a strain overexpressing the ε subunit and a strain harboring an engineered mutation in BDQ’s ε subunit binding site suggest that TBAJ-876 retains BDQ’s activity on the ε subunit. Nuclear magnetic resonance (NMR) titration studies confirmed that TBAJ-876 binds to the ε subunit at BDQ’s binding site. We show that TBAJ-876 retains BDQ’s antimycobacterial mode of action. The developmental compound inhibits the mycobacterial F-ATP synthase via a dual-subunit mechanism of interfering with the functions of both the enzyme’s c and ε subunits.

## Abstract (precis)

The antituberculosis drug bedaquiline (BDQ) inhibits Mycobacterium tuberculosis F-ATP synthase by interfering with two subunits. Drug binding to the c subunit stalls the rotation of the c ring, while binding to the ε subunit blocks coupling of c ring rotation to ATP synthesis at the catalytic α_3_:β_3_ headpiece. BDQ is used for the treatment of drug-resistant tuberculosis.

## INTRODUCTION

The mycobacterial F-ATP synthase is composed of two parts, a membrane-embedded F_O_ domain and a cytoplasmic F_1_ domain (1, 2). The F_O_ domain is made up of subunits a and b and the c ring (composed of nine c subunits [3]), while the F_1_ domain is made up of subunits γ, δ, and ε and the α_3_:β_3_ headpiece (4). The α_3_:β_3_ headpiece is connected to the c ring via a central stalk composed of subunits ε and γ and a peripheral stalk composed of subunits b and δ (2). Proton translocation from the periplasmic space to the cytoplasmic side is associated with rotation of the c ring. This rotation is transmitted by the ε and γ subunits to the catalytic α_3_:β_3_ headpiece, where it drives conformational changes powering ATP synthesis (2–6).

**FIG 1:**
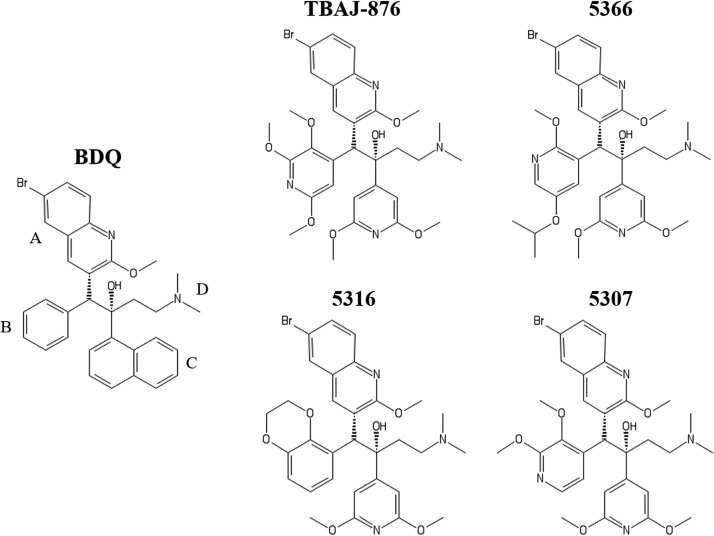
Structures of BDQ and TBAJ-876 and its analogues. TBAJ-876, 5366, 5316, and 5307 have been described previously (20), with TBAJ-876 corresponding to “compound 46,” 5366 to “compound 29,” 5316 to “compound 17,” and 5307 to “compound 33.” BDQ’s quinoline (A) and dimethylamino (D) groups are retained in the compounds, while its phenyl (B) and naphthalene (C) groups are replaced.

Bedaquiline (BDQ) (Sirturo) (Fig. 1) is a first-in-class diarylquinoline antituberculosis (anti-TB) drug that functions by inhibiting mycobacterial F-ATP synthase (7). Isolation and characterization of BDQ-resistant Mycobacterium tuberculosis mutants followed by biochemical, biophysical, and computational studies showed that BDQ inhibits the F-ATP synthase by binding to the c subunit (3, 7–10). Drug binding presumably results in stalling of c ring rotation, which prevents ATP synthesis at the α_3_:β_3_ headpiece (3). Biophysical studies revealed that BDQ has a second binding site on the mycobacterial F-ATP synthase, around the W16 amino acid residue of the ε subunit (6, 11). Drug susceptibility studies employing a Mycobacterium smegmatis strain overexpressing the ε subunit and an engineered M. smegmatis strain harboring a mutation of the ε subunit W16 residue provided evidence that the drug interacts with the ε subunit *in vivo* (12). However, spontaneous BDQ-resistant mutations in the ε subunit could not be isolated (10), suggesting that the ε subunit binding site of BDQ does not tolerate resistance-conferring missense mutations without affecting the viability of the bacterium. BDQ binding to the ε subunit has been proposed to interfere with the subunit’s coupling function of communicating c ring rotation to the catalytic α_3_:β_3_ headpiece (6).

BDQ has pharmacological and toxicological liabilities. The drug is highly lipophilic (cLogP = 7.25); is associated with prolongation of the QT interval due to inhibition of cardiac human ERG (hERG) potassium ion channels; and has a very long terminal half-life, leading to concerns regarding tissue accumulation (13–15). Recent medicinal chemistry campaigns resulted in the discovery of 3,5-dialkoxypyridine analogues of BDQ that address these issues (16–20). A new developmental compound of this series, TBAJ-876 (Fig. 1), not only shows improved physicochemical properties and less inhibition of the hERG channel, it is also more potent against M. tuberculosis and retains efficacy in a mouse model of TB (20).

Here, we asked whether TBAJ-876 retains the antimycobacterial mechanism of action of BDQ. In addition to TBAJ-876, three of its analogues (5366, 5316, and 5307) (Fig. 1) were also characterized to provide evidence that the results hold true for the series. Biochemical, genetic, and biophysical analyses suggest that TBAJ-876 inhibits the mycobacterial F-ATP synthase similarly to BDQ by a dual mechanism of interfering with the functions of both the enzyme’s c and ε subunits.

## RESULTS

### TBAJ-876 inhibits ATP synthesis by the mycobacterial F-ATP synthase and depletes intrabacterial ATP.

**FIG 2:**
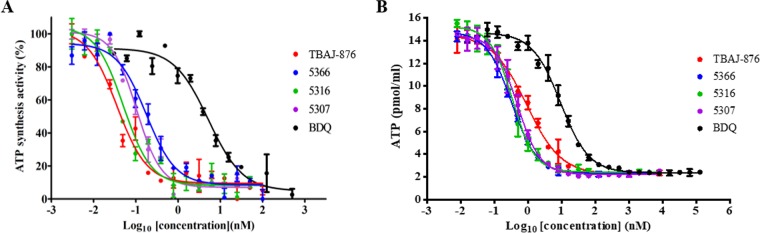
Effects of TBAJ-876 and its analogues on ATP synthesis in M. bovis BCG. Shown are the effects of the compounds on ATP synthesized by M. bovis BCG inverted membrane vesicles (A) and the intrabacterial ATP content of whole-cell M. bovis BCG treated for 24 h (B). BDQ was used as a positive control. The IC_50_ values are presented in Table 1. (A) All the values are represented as percentages of the ATP synthesized by the drug-free sample (4.22 nmol/mg of protein). (B) The *y* axis shows the amounts of ATP per milliliter of culture. The numbers of CFU of all the samples were constant at the end of treatment compared to the start of the experiment. Experiments were carried out thrice independently. The results are shown as mean values with standard deviations.

To determine whether TBAJ-876 inhibits ATP synthesis catalyzed by the mycobacterial F-ATP synthase, biochemical assays employing inverted membrane vesicles prepared from Mycobacterium bovis BCG were carried out. TBAJ-876 and its analogues inhibited ATP synthesis by the inverted vesicles with 50% inhibitory concentration (IC_50_) values ranging from 0.031 to 0.2 nM (Fig. 2A and Table 1). In contrast, BDQ inhibited ATP synthesis by the inverted vesicles with an IC_50_ value of 5.3 nM (Fig. 2A and Table 1). These results show that TBAJ-876 and its analogues inhibit mycobacterial ATP synthesis and have greater potency against the mycobacterial F-ATP synthase than BDQ.

**TABLE 1:** Potencies of TBAJ-876 and its analogues in inhibiting ATP synthesis by the F-ATP synthase, depleting intrabacterial ATP, and inhibiting growth of M. bovis BCG

Compound	IC_50_ (nM)^*a*^		Growth inhibition MIC_90_ (nM)^*b*^
	ATP synthesis	ATP depletion	
TBAJ-876	0.031	0.89	7.2
5366	0.200	0.32	3.4
5316	0.047	0.34	3.6
5307	0.091	0.48	3.6
BDQ	5.300	9.16	70

a The IC_50_ values are derived from Fig. 2. The order of the ATP synthesis IC_50_ values is TBAJ-876 < 5316 < 5307 < 5366 ≪ BDQ. The order of the intrabacterial ATP depletion IC_50_ values is 5366 < 5316 < 5307 < TBAJ-876 ≪ BDQ.

b MIC_90_ is the MIC required to inhibit 90% of M. bovis BCG growth. The experiment was carried out thrice independently, and the results shown are mean values. The order of the MIC_90_ values is 5366 < 5316 and 5307 < TBAJ-876 ≪ BDQ.

If inhibition of the mycobacterial F-ATP synthase is the antimycobacterial mechanism of action of TBAJ-876, treatment of intact bacteria should result in depletion of the intrabacterial ATP content. To test this prediction, M. bovis BCG cultures were treated with TBAJ-876 and its analogues, and the intrabacterial ATP content was measured. The compounds indeed reduced ATP levels, with IC_50_ values ranging from 0.32 to 0.89 nM (Fig. 2B and Table 1). In contrast, BDQ reduced ATP levels with an IC_50_ value of 9.16 nM (Fig. 2B and Table 1). These results show that TBAJ-876 and its analogues were more potent than BDQ in depleting ATP content, consistent with their greater potency on the enzyme (Fig. 2A and Table 1). In line with their greater potencies in the ATP synthesis and depletion assays, these new compounds displayed lower MICs than BDQ against M. bovis BCG (Table 1). Furthermore, the order of the intrabacterial ATP depletion IC_50_ values of the compounds and BDQ is similar to that of the MIC values (Table 1), suggesting a correlation between intrabacterial ATP depletion and growth inhibition.

Inhibition of ATP synthesis was also assessed for the fast-growing model organism M. smegmatis. TBAJ-876 and its analogues inhibited ATP synthesis by M. smegmatis inverted membrane vesicles and caused a decrease of intrabacterial ATP content in M. smegmatis cultures (see Fig. S1A and B in the supplemental material). Again, the potencies of the compounds on the M. smegmatis F-ATP synthase were higher than those of BDQ (see Fig. S1A). These results suggest that targeting the mycobacterial F-ATP synthase by TBAJ-876 and its analogues is conserved across mycobacteria.

### TBAJ-876 inhibits mycobacterial F-ATP synthase’s ATP hydrolysis activity.

Since BDQ inhibits not only ATP synthesis but also ATP hydrolysis, TBAJ-876 and its analogues were assessed for the ability to inhibit the latter in M. smegmatis inverted membrane vesicles using a regeneration assay (21). As presented in Fig. S1C, BDQ inhibited ATPase activity by 78% at 100 μM and almost completely at 250 μM. TBAJ-876, 5316, and 5307 showed BDQ-like “strong” inhibition of ATPase activity (see Fig. S1C). However, 5366 reduced ATPase activity by only 36% at 100 μM and 40% at 250 μM (see Fig. S1C). These results suggest that, like BDQ, TBAJ-876 and its analogues also inhibit mycobacterial F-ATP synthase in its hydrolysis mode. Interestingly, inhibition of hydrolysis activity appears to occur in a compound-specific manner.

### TBAJ-876 targets the c subunit of the mycobacterial F-ATP synthase via BDQ’s binding site.

BDQ’s targeting of the mycobacterial F-ATP synthase c subunit was initially determined via isolation and characterization of BDQ-resistant M. tuberculosis mutants. This uncovered the fact that resistance to BDQ is associated with missense mutations at the c subunit’s amino acid residues D28, E61, A63, and I66 (9, 10). Subsequent structural analyses using the c subunit of Mycobacterium phlei showed that counterparts of these amino acid residues form the binding site of BDQ (3) (see Fig. S2 in the supplemental material). To determine whether TBAJ-876 and its analogues retain BDQ’s targeting of the c subunit, M. tuberculosis spontaneous resistant mutants were selected against the compounds. Similar to BDQ (7, 10), resistant mutants arose at a low frequency of around 10^−8^ to 10^−9^/CFU (Table 2), and targeted sequencing of the *atpE* gene (encoding the c subunit) of the mutants revealed missense mutations at the same amino acid positions observed previously for BDQ (Table 2). MIC determinations of the resistant mutants showed high levels of resistance (>40-fold MIC increase) to TBAJ-876 and its analogues (Table 3). Furthermore, these resistant mutants were cross-resistant to BDQ (Table 3). Taken together, these results suggest that TBAJ-876 and its analogues target the c subunit of the mycobacterial F-ATP synthase by binding to the subunit’s BDQ binding site.

**TABLE 2:** Genotypic characteristics of spontaneous M. tuberculosis mutants resistant to TBAJ-876 and its analogues^*a*^

Compound	Mutation frequency^*b*^	No. of *atpE* mutants/total no. of mutants isolated^*c*^	Missense mutation (DNA/amino acid) in *atpE* (no. of mutants with the particular mutation)
TBAJ-876	4.23 × 10^−9^	5/5	A83C/D28A (1)
			G183T/E61D (1)
			G187C/A63P (3)
5366	2.54 × 10^−8^	28/28	A83T/D28V (2)
			A83G/D28G (6)
			G183T/E61D (1)
			G187C/A63P (19)
5316	6.36 × 10^−9^	7/7	A83G/D28G (1)
			A83C/D28A (3)
			A83T/D28V (3)
5307	3.00 × 10^−8^	33/34	A83C/D28A (4)
			A83G/D28G (4)
			A83T/D28V (8)
			G183T/E61D (7)
			G187C/A63P (8)
			C198G/I66M (2)

a Isolation of spontaneous resistant mutants was carried out twice independently.

b The mutation frequencies displayed are mean values of two biological replicates.

c The total number of mutants represents the total number isolated across two biological replicates. In addition to the *atpE* gene, the *atpC* genes of all the mutants were sequenced. No polymorphisms were observed in the *atpC* gene.

**TABLE 3:** Levels of resistance of c subunit mutant M. tuberculosis strains to TBAJ-876 and its analogues^*a*^

Compound	M. tuberculosis H37Rv MIC_90_ (nM)^*b*^	MIC_90_ fold increase in M. tuberculosis c subunit mutant strains^*c*^					
		D28A	D28G	D28V	E61D	A63P	I66M
TBAJ-876	125	140	104	132	100	48	59
5366	30	333	300	250	45	50	50
5316	70	200	200	200	105	72	42
5307	70	357	235	360	100	150	87
BDQ	400	>400	>400	>400	312	>400	262

a The experiment was carried out thrice independently, and BDQ was used as a positive control.

b The MIC_90_ values displayed are mean values of three biological replicates.

c The mutant strains are labeled by the missense mutation that is present in the c subunit of the strain.

### TBAJ-876 targets the ε subunit of F-ATP synthase *in vivo* via BDQ’s binding site.

While BDQ resistance mutations in the *atpE* gene can be isolated, such mutations in the *atpC* gene (encoding the ε subunit) have not been obtained (10). Similarly, in the current work, identified spontaneous resistant mutations mapped only to the *atpE* gene. Resistance mutations in *atpC* were not uncovered (the spontaneous resistance mutation frequency for *atpC* was <10^−9^/CFU [Table 2]). To determine whether TBAJ-876 has retained BDQ’s targeting of the ε subunit, we employed a strategy we used previously to demonstrate *in vivo* interaction of BDQ with the ε subunit (12). This involves susceptibility testing of an ε subunit overexpresser strain of M. smegmatis and of an M. smegmatis strain harboring an engineered mutation at the W16 residue, which resides at BDQ’s binding site (12). Previously, we showed that constitutive overexpression of the ε subunit decreased susceptibility to BDQ, plausibly by sequestration of the drug (12). We also showed that replacing W16 with an alanine via site-directed genome mutagenesis caused increased susceptibility to BDQ, possibly by allowing tighter binding of the drug to its binding site (12). Here, we show that ε subunit overexpression decreased susceptibility and that the W16A mutation also increased susceptibility to TBAJ-876 and its three analogues (Table 4). These results indicate that TBAJ-876 and its analogues target the ε subunit of mycobacterial F-ATP synthase *in vivo* via BDQ’s binding site around the W16 residue.

**TABLE 4:** Effects of ε subunit overexpression and ε subunit W16A missense mutation on M. smegmatis susceptibility to TBAJ-876 and its analogues^*a*^

Compound	MIC_50_ ± SD (nM)^*b*^		
	M. smegmatis WT	ε overexpresser	W16A
TBAJ-876	1.1 ± 0.05	2.75 ± 0.02	0.10 ± 0.002
5366	2.1 ± 0.06	14.3 ± 0.07	0.30 ± 0.02
5316	0.6 ± 0.03	1.68 ± 0.02	0.14 ± 0
5307	0.8 ± 0.05	8 ± 0.10	0.14 ± 0.038
BDQ	12 ± 0.10	24 ± 0.10	4 ± 0.15

a The experiment was carried out thrice independently. BDQ was used as a positive control.

b The results shown are mean values with standard deviations (SD). MIC_50_ is the MIC required to inhibit 50% of the growth of the M. smegmatis strain. WT, wild type.

### TBAJ-876 binds to BDQ’s binding site on the ε subunit *in vitro*.

**FIG 3:**
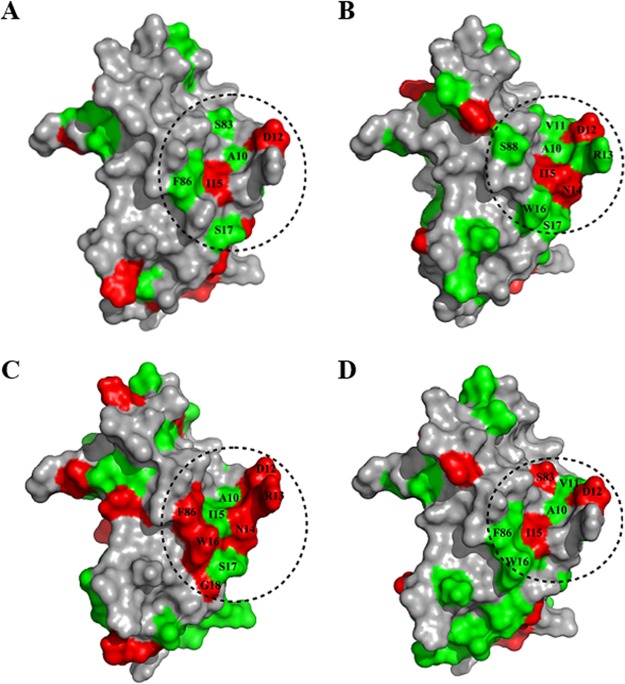
Structural effects of binding of TBAJ-876 and its analogues to the M. tuberculosis ε subunit based on NMR titration. Amino acid residues that were perturbed due to the binding of TBAJ-876 (A), 5366 (B), 5316 (C), and 5307 (D) are colored and mapped onto the previously reported surface of the M. tuberculosis ε subunit (6). Residues having CSP values higher than 0.02 ppm are represented in red, while the residues with CSP values between 0.01 and 0.02 ppm are represented in green. The regions of the M. tuberculosis ε subunit inside the dashed circles represent BDQ’s previously reported binding sites (6). Amino acid residues within the binding site that were perturbed are labeled by their one-letter codes.

The binding of BDQ to the isolated M. tuberculosis ε subunit has been previously determined via nuclear magnetic resonance (NMR) titration using a highly resolved and dispersed NMR spectrum of the protein (6). To confirm that TBAJ-876 and its analogues bind to BDQ’s binding site around the W16 amino acid residue, as suggested by the drug susceptibility shift observed for the M. smegmatis W16A mutant strain, we carried out NMR titration experiments. Binding of the new compounds to the M. tuberculosis ε subunit induced significant chemical shift perturbations (CSPs) (Fig. 3; see Fig. S3 in the supplemental material), most prominently at the A10-to-W16 amino acid region, the binding site of BDQ (see Fig. S2) (6). This result suggests that TBAJ-876, its analogues, and BDQ all share the same binding site on the M. tuberculosis ε subunit.

Significant CSPs were also observed in additional specific regions of the M. tuberculosis ε subunit (Fig. 3; see Fig. S3). Similar to BDQ binding, binding of TBAJ-876 and its analogues induced structural alterations in the M1-to-N5, L41-to-V46, and R62-to-E74 amino acid regions of the N-terminal domain and the D91-to-I120 amino acid region of the C-terminal domain of the protein (Fig. 3; see Fig. S3). These binding-induced intraprotein structural changes have been previously proposed to corrupt the ε subunit’s coupling function by affecting interdomain communication (6). Thus, TBAJ-876 and its analogues appear not only to share BDQ’s binding site on the ε subunit, but also to induce BDQ-like coupling-disrupting structural changes inside the protein.

## DISCUSSION

Recent systematic medicinal chemistry programs have delivered promising candidates for the development of the next-generation BDQ with improved physicochemical, pharmacological, and safety properties (16–20). One of these candidates, TBAJ-876, is a 3,5-dialkoxypyridine analogue of BDQ that is less lipophilic and less cardiotoxic and shows higher clearance while retaining efficacy in mice similar to that of the parental drug (20). This analogue has advanced to preclinical development (20). In this study, we addressed the question of whether BDQ’s on-target activity is retained by TBAJ-876 by studying the mechanism of action of the compound and three of its analogues.

We show that the compounds retain BDQ’s ability to inhibit the mycobacterial F-ATP synthase in both its synthesis and hydrolysis modes (Fig. 2A; see Fig. S1A and C). Furthermore, the compounds are more potent than the parental drug in inhibiting ATP synthesis by the enzyme and depleting intrabacterial ATP content (Fig. 2 and Table 1; see Fig. S1A). These increased potencies correlate with the lower MICs of the compounds against M. bovis BCG (Table 1) and M. tuberculosis (Table 3) compared to BDQ.

After showing that TBAJ-876 and its analogues retained targeting of the mycobacterial F-ATP synthase, we next determined whether the compounds also retained BDQ’s dual on-target mechanism, namely, interfering with the c subunit and the ε subunit of the enzyme. We confirmed that TBAJ-876 and its analogues target the c subunit, as evidenced by the isolation of spontaneous resistance mutations in the *atpE* gene (Table 2). The findings that the obtained missense mutations were at BDQ’s binding site (see Fig. S2) (3) and confer high levels of resistance to the analogues, as well as cross-resistance to BDQ (Table 3), suggest that the new compounds share the same binding site on the c subunit as BDQ. Thus, as proposed for BDQ, binding of TBAJ-876 and its analogues to this binding site may stall c ring rotation and prevent the flow of protons down the transmembrane proton gradient, consequently inhibiting ATP synthesis at the catalytic α_3_:β_3_ headpiece of the enzyme (3).

The BDQ-like susceptibility shifts of TBAJ-876 and its analogues in engineered mutant ε subunit M. smegmatis strains, either overexpressing the ε subunit or harboring a W16A mutation, provide *in vivo* evidence that the compounds retain BDQ’s targeting of the ε subunit via the region around the W16 amino acid residue (Table 4). Through characterizing the interaction of TBAJ-876 and its analogues with the M. tuberculosis ε subunit *in vitro* via NMR titration, we show that the compounds induce structural changes prominently in the A10-to-W16 amino acid region (Fig. 3; see Fig. S3). Since this amino acid region represents BDQ’s binding site on the ε subunit (see Fig. S2) (6), these *in vitro* data, together with our *in vivo* findings, collectively suggest that TBAJ-876 and its analogues share the same binding site on the ε subunit as BDQ.

The NMR titration data also revealed that binding of TBAJ-876 and its analogues induces specific structural changes throughout the N- and C-terminal domains of the ε subunit (Fig. 3; see Fig. S3). Interestingly, these intraprotein structural changes were similar to structural changes previously observed upon BDQ binding to the subunit (6). For BDQ, it was argued that these drug-induced intraprotein alterations affect the interdomain amino acid interaction network required for M. tuberculosis ε subunit’s coupling function (6). Thus, TBAJ-876 and its analogues appear not only to bind to the same site on the ε subunit as BDQ, but also to induce BDQ-like coupling-disrupting structural changes inside the protein.

Our *in vivo* and *in vitro* analyses of the interaction of TBAJ-876 and its analogues with the ε subunit suggest that the compounds target the subunit. However, similar to BDQ (10), attempts to isolate spontaneous resistance mutations in the ε subunit were not productive. The mechanism of action and resistance to another F-ATP synthase inhibitor, *N*,*N*′-dicyclohexylcarbodiimide (DCCD), provide an interesting parallel to this finding. DCCD targets the c subunit (22–24) and the β subunit of the enzyme (25, 26). Interestingly, DCCD has been shown in M. phlei to bind to a binding site on the c subunit similar to that of BDQ (3). Analyses of DCCD-resistant strains of Escherichia coli (24) and Streptococcus faecalis (22) have revealed that DCCD resistance mutations can be isolated only in the c subunit, but not in the β subunit. However, a mutational study of the binding site of DCCD on the β subunit of E. coli (residue E181) has shown that site-directed mutagenesis of this key residue results in impairment of the catalytic activity of the subunit (27). This suggests that DCCD resistance mutations in the β subunit are not tolerated by the bacteria due to their deleterious effect on F-ATP synthase activity. This may also be the case for BDQ and TBAJ-876 with regard to the ε subunit. Biochemical studies on the engineered M. smegmatis ε subunit W16A mutant strain show that the mutation causes a strong reduction of ATP synthesis by the mycobacterial F-ATP synthase (6). This appears to be caused by a destabilizing effect of the amino acid exchange on the interdomain amino acid interaction network that is crucial for the ε subunit’s coupling function (6). Thus, missense mutations in the BDQ/TBAJ-876 binding site on the ε subunit may not be tolerated, providing a plausible explanation for the inability to isolate such mutations.

**FIG 4:**
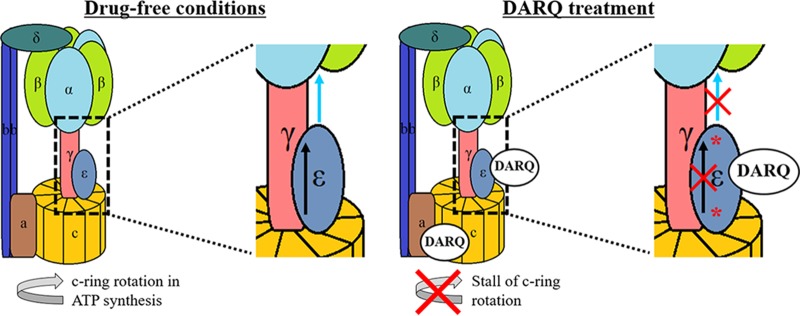
Model of the activities of BDQ and TBAJ-876 on the mycobacterial F-ATP synthase. During ATP synthesis under drug-free conditions, the c ring rotates and the ε subunit interacts with the α_3_:β_3_ headpiece to communicate this rotational movement as part of its coupling function. The coupling function of the ε subunit is carried out by the transfer of information on c ring rotation, in the form of conformational alterations, from the N-terminal domain to the C-terminal domain through an interdomain amino acid interaction network (represented by the black arrows) (6). The communication to the α_3_:β_3_ headpiece is complete when the C-terminal domain of the ε subunit adopts an extended conformation to interact with the α_3_:β_3_ headpiece (represented by the blue arrows) (6). BDQ or TBAJ-876 (represented by “DARQ” [diarylquinoline]) binds to two sites on the F-ATP synthase: the c subunit and the ε subunit. Binding to the c subunit has been proposed to stall c ring rotation, which inhibits ATP synthesis at the α_3_:β_3_ headpiece (3). Binding to the ε subunit induces structural changes throughout its N- and C-terminal domains (asterisks) (6). These intraprotein structural changes have been proposed to corrupt the interdomain interaction network, thus preventing the transfer of information on c ring rotation. Hence, the ε subunit’s coupling function of communicating c ring rotational movement to the α_3_:β_3_ headpiece is corrupted, and consequently, ATP synthesis is inhibited (6).

In conclusion, we show that TBAJ-876 and three of its analogues retain BDQ’s antimycobacterial mode of action. This new developmental compound inhibits the mycobacterial F-ATP synthase via a dual-subunit on-target mechanism of interfering with the functions of the enzyme’s c and ε subunits (Fig. 4).

## MATERIALS AND METHODS

### Bacterial strains, culture medium, and chemicals.

M. bovis BCG (ATCC 35734), M. smegmatis mc^2^ 155 (ATCC 700084), and M. tuberculosis H37Rv (ATCC 27294) wild-type and derived mutant strains were maintained in complete Middlebrook 7H9 medium (BD Difco) supplemented with 0.5% (vol/vol) glycerol (Fisher Scientific), 0.05% (vol/vol) Tween 80 (Sigma-Aldrich), and 10% (vol/vol) Middlebrook albumin-dextrose-catalase (ADC) (BD Difco). For culturing of the M. smegmatis ε subunit overexpresser mutant strain, kanamycin (Sigma-Aldrich) was added to the culture medium to create a final concentration of 25 μg/ml to serve as a selection antibiotic for the episomal pMV262 plasmid harbored by the strain. BDQ was purchased from MedChem Express, freshly dissolved in 100% dimethyl sulfoxide (DMSO) to create a concentration of 0.5 mM, and sterilized using 0.2-μm polytetrafluoroethylene (PTFE) membrane filters (Acrodisc; Pall). TBAJ-876, 5366, 5316, and 5307 were described previously (20), with TBAJ-876 corresponding to “compound 46,” 5366 to “compound 29,” 5316 to “compound 17,” and 5307 to “compound 33.” TBAJ-876 and its analogues were dissolved in 100% DMSO to create a concentration of 50 mM. All the chemicals used for the ATP synthesis assay, ATP hydrolysis assay, and NMR titration were purchased from Biomol, Merck, Sigma, or Serva unless otherwise stated.

### Preparation of M. bovis BCG and M. smegmatis inverted membrane vesicles.

Inverted membrane vesicles were prepared according to a method described previously (6). Briefly, the mycobacterial wild-type cells were lysed and subsequently broken at high pressure. The membrane fraction was then extracted and pelleted to obtain the vesicles.

### ATP synthesis assay.

The reaction mixture used for the ATP synthesis assay contained assay buffer (50 mM MOPS [morpholinepropanesulfonic acid]-NaOH, 10 mM MgCl_2_, pH 7.5), 10 μM ADP, 250 μM P_i_, and 1 mM NADH. KH_2_PO_4_ salt (100 mM) was dissolved in the assay buffer to adjust the concentration of P_i_; 25 μl of the reaction mixture was added to each well of an opaque, white, 96-well, flat-bottom Nunc plate (Thermo Scientific). Each compound or BDQ was added to the first well of each row to create two times the desired highest final concentration. A 19-point 2-fold serial dilution was then carried out starting from the first well. The M. bovis BCG or M. smegmatis inverted membrane vesicles were then added to create a final concentration of 5 μg of protein/ml in 50 μl per well. Subsequently, the plate was incubated at room temperature for 30 min. At the end of the incubation period, 50 μl of CellTitre-Glo (Promega) was added to each well. The plate was then incubated again for 10 min in the dark at room temperature. Subsequently, luminescence was measured with a Tecan Infinite Pro 200 plate reader (parameters: luminescence, integration time of 500 ms; no attenuation). The level of luminescence correlates with the amount of ATP synthesized by the F-ATP synthase. The graphs of the results were made using GraphPad Prism 5 software.

### Quantification of intrabacterial ATP.

Clear 96-well flat-bottom Costar cell culture plates (Corning) were filled with 100 μl of complete 7H9 medium in each well. Each compound or BDQ was added to the first well in each row to create two times the desired highest final concentration. Subsequently, a 21-point 2-fold serial dilution was carried out starting from the first well in each row. M. bovis BCG, which was grown to mid-exponential phase, was diluted to an optical density at 600 nm (OD_600_) of 0.1; 100 μl of the diluted culture was added to each well to create a final OD_600_ of 0.05 in all the wells. The plates were incubated at 37°C on an orbital shaker set at 110 rpm for 24 h.

For the assay involving M. smegmatis, the strain was grown to mid-exponential phase and subsequently diluted to an OD_600_ of 0.05; 1 ml of diluted culture was transferred to each 14-ml round-bottom tube (SPL Life Sciences). The compounds and BDQ were added to their respective tubes to achieve the desired final concentrations. The tubes were incubated at 37°C with shaking at 160 rpm for 3 h.

At the end of the incubation period for both M. bovis BCG and M. smegmatis samples, the samples were measured for their intrabacterial ATP content by employing the BacTitre-Glo microbial cell viability assay (Promega), which was carried out according to the manufacturer’s instructions as described previously (28). Twenty-five microliters of each sample was mixed with 25 μl of the BacTitre-Glo reagent in each well of an opaque, white, 96-well, flat-bottom Nunc plate. Luminescence was measured with a Tecan Infinite Pro 200 plate reader after 5 min of incubation of the plate in the dark at room temperature. The background luminescence reading was subtracted from the luminescence readings of all the samples. The ATP amount was derived from the respective luminescence readings using a standard curve of a range of known ATP amounts. The graph of the results was made using GraphPad Prism 5 software.

### ATP hydrolysis assay.

The effects of the compounds and BDQ on the ATPase activity of M. smegmatis inverted membrane vesicles was measured using a continuous ATP hydrolysis assay as described previously (6), but with a minor modification. The modification to the assay was that at the 30-s mark of the 5-min observation period, the compounds or BDQ was added to the reaction mixture to achieve their respective tested concentrations. Observation of absorbance continued subsequently to assess the effects of the compounds and BDQ on the absorbance. The graph of the results was made using GraphPad Prism 5 software.

### Selection of spontaneous resistant mutants.

Mid-exponential-phase cultures (10^7^ and 10^8^ CFU) of M. tuberculosis H37Rv were plated out on Middlebrook 7H10 agar plates supplemented with 0.5% (vol/vol) glycerol and 10% (vol/vol) Middlebrook oleic acid-albumin-dextrose-catalase (OADC) (BD Difco) and containing 5×, 10×, 20×, 40×, or 80× the MIC_90_ of each compound (the MIC_90_ values are shown in Table 3). The agar plates were incubated at 37°C for 4 weeks, and the colonies that grew on the plates were restreaked onto agar plates containing the same concentration of the respective compound to confirm resistance. All the colonies from the restreak plates were picked and expanded in complete 7H9 medium to an OD_600_ of 1.0. Subsequently, the cultures were transferred to 50-ml Falcon tubes (Fisher Scientific) and centrifuged at 3,200 rpm at 25°C for 10 min. The supernatant was discarded, and the cell pellet was suspended in an equal volume of 7H9 supplemented with 10% (vol/vol) glycerol, 0.05% (vol/vol) Tween 80, and 10% (vol/vol) ADC. The mutants were stored as 1-ml aliquots at −80°C. The frozen stocks were used for genetic and phenotypic characterization of the mutants.

### Sequencing of *atpC* and *atpE* genes.

Genomic DNA of the M. tuberculosis spontaneous resistant mutants was isolated as described previously (29). Briefly, the cells were lysed, broken up via bead beating, and delipidated. Subsequently, the genomic DNA was isolated via precipitation. PCR amplification of the entire F-ATP synthase operon from the genomic DNA of all the mutants was first carried out using the ATPoperon Frwd and ATPoperon Rev primers (see Table S1 in the supplemental material). The amplified PCR product of the F-ATP synthase operon, which functioned as a template, and the respective gene-specific sequencing primers (see Table S1) were then used for sequencing the *atpC* and *atpE* genes (encoding the ε subunit and c subunit, respectively). Sequencing was carried out by Bio Basic Inc. (Singapore). The sequences of the two genes of all the mutants were compared to those of the wild-type strain to identify mutations.

### NMR titration of the compounds with the M. tuberculosis ε subunit.

All samples used for the NMR titration experiment contained 0.2 mM ^15^N-labeled M. tuberculosis ε subunit in 50 mM Tris, pH 7.5, 200 mM NaCl, 10% (vol/vol) glycerol, 0.01% (vol/vol) NaN_3_, and 10% (vol/vol) D_2_O. NMR titration was carried out by titrating each compound stepwise into the sample solution to create a final molar ratio (protein/compound) of 1:2. The NMR measurements were carried out using a Bruker Avance 700-MHz spectrometer that was equipped with a cryogenic probe at 293 K. Resonance assignments of ^15^N and ^1^H resonances were carried out based on the previously reported NMR solution structure of the M. tuberculosis ε subunit (Protein Data Bank ID 5YIO). All NMR spectra were processed using NMRPipe (30) and subsequently analyzed using SPARKY (31).

### M. smegmatis ε subunit mutant strains.

The M. smegmatis ε subunit W16A mutant strain and the M. smegmatis ε subunit overexpresser strain were described previously (12).

### Growth inhibition dose-response assay.

The growth inhibition dose-response assay was carried out using the broth microdilution method as described previously (32). Briefly, each well of clear 96-well flat-bottom Costar cell culture plates (Corning) was filled with 100 μl of complete 7H9 medium. Each compound or BDQ was added to the first well in each row to create two times the desired highest final concentration. Subsequently, a 10-point 2-fold serial dilution was carried out starting from the first well in each row. The mycobacterial strains used for the assay were grown to mid-exponential phase and subsequently diluted to an OD_600_ of 0.1; 100 μl of the diluted culture was added to each well to create a final OD_600_ of 0.05 in all the wells. For M. bovis BCG, the plates were incubated at 37°C on an orbital shaker set at 110 rpm for 5 days. For M. smegmatis strains, the plates were incubated at 37°C on an orbital shaker set at 110 rpm for 1 day. For M. tuberculosis strains, the plates were incubated at 37°C on an orbital shaker set at 80 rpm for 7 days. At the end of the incubation period, the cultures in all the wells were manually resuspended, and the OD_600_ was read using a Tecan Infinite Pro 200 plate reader. The MIC_50_ reported represents the concentration that inhibited 50% of growth compared to the untreated culture. The MIC_90_ reported represents the concentration that inhibited 90% of growth compared to the untreated culture.

## Supplementary Material

Supplemental file 1

## References

[1] Lu P, Lill H, Bald D 2014 ATP synthase in mycobacteria: special features and implications for a function as drug target. Biochim Biophys Acta 1837:1208–1218. doi:10.1016/j.bbabio.2014.01.022. 24513197

[2] Kamariah N, Huber RG, Nartey W, Bhushan S, Bond PJ, Grüber G 2019 Structure and subunit arrangement of mycobacterial F1FO ATP synthase and novel features of the unique mycobacterial subunit δ. J Struct Biol 207:199–208. doi:10.1016/j.jsb.2019.05.008. 31132404

[3] Preiss L, Langer JD, Yildiz O, Eckhardt-Strelau L, Guillemont JE, Koul A, Meier T 2015 Structure of the mycobacterial ATP synthase Fo rotor ring in complex with the anti-TB drug bedaquiline. Sci Adv 1:e1500106. doi:10.1126/sciadv.1500106. 26601184 PMC4640650

[4] Zhang AT, Montgomery MG, Leslie AG, Cook GM, Walker JE 2019 The structure of the catalytic domain of the ATP synthase from Mycobacterium smegmatis is a target for developing antitubercular drugs. Proc Natl Acad Sci U S A 116:4206–4211. doi:10.1073/pnas.1817615116. PMC6410841 30683723

[5] Diez M, Zimmermann B, Börsch M, König M, Schweinberger E, Steigmiller S, Reuter R, Felekyan S, Kudryavtsev V, Seidel CAM, Gräber P 2004 Proton-powered subunit rotation in single membrane-bound F 0 F 1-ATP synthase. Nat Struct Mol Biol 11:135–141. doi:10.1038/nsmb718. 14730350

[6] Joon S, Ragunathan P, Sundararaman L, Nartey W, Kundu S, Manimekalai MSS, Bogdanović N, Dick T, Grüber G 2018 The NMR solution structure of Mycobacterium tuberculosis F-ATP synthase subunit epsilon provides new insight into energy coupling inside the rotary engine. FEBS J 285:1111–1128. doi:10.1111/febs.14392. 29360236

[7] Andries K, Verhasselt P, Guillemont J, Gohlmann HW, Neefs JM, Winkler H, Van Gestel J, Timmerman P, Zhu M, Lee E, Williams P, de Chaffoy D, Huitric E, Hoffner S, Cambau E, Truffot-Pernot C, Lounis N, Jarlier V 2005 A diarylquinoline drug active on the ATP synthase of Mycobacterium tuberculosis. Science 307:223–227. doi:10.1126/science.1106753. 15591164

[8] Petrella S, Cambau E, Chauffour A, Andries K, Jarlier V, Sougakoff W 2006 Genetic basis for natural and acquired resistance to the diarylquinoline R207910 in mycobacteria. Antimicrob Agents Chemother 50:2853–2856. doi:10.1128/AAC.00244-06. 16870785 PMC1538646

[9] Koul A, Dendouga N, Vergauwen K, Molenberghs B, Vranckx L, Willebrords R, Ristic Z, Lill H, Dorange I, Guillemont J, Bald D, Andries K 2007 Diarylquinolines target subunit c of mycobacterial ATP synthase. Nat Chem Biol 3:323–324. doi:10.1038/nchembio884. 17496888

[10] Huitric E, Verhasselt P, Koul A, Andries K, Hoffner S, Andersson DI 2010 Rates and mechanisms of resistance development in Mycobacterium tuberculosis to a novel diarylquinoline ATP synthase inhibitor. Antimicrob Agents Chemother 54:1022–1028. doi:10.1128/AAC.01611-09. 20038615 PMC2825986

[11] Biuković G, Basak S, Manimekalai MS, Rishikesan S, Roessle M, Dick T, Rao SP, Hunke C, Grüber G 2013 Variations of subunit ε of the Mycobacterium tuberculosis F1Fo ATP synthase and a novel model for mechanism of action of the tuberculosis drug TMC207. Antimicrob Agents Chemother 57:168–176. doi:10.1128/AAC.01039-12. 23089752 PMC3535943

[12] Kundu S, Biuković G, Grüber G, Dick T 2016 Bedaquiline targets the epsilon subunit of mycobacterial F-ATP Synthase. Antimicrob Agents Chemother 60:6977–6979. doi:10.1128/AAC.01291-16. 27620476 PMC5075122

[13] Diacon AH, Donald PR, Pym A, Grobusch M, Patientia RF, Mahanyele R, Bantubani N, Narasimooloo R, De Marez T, van Heeswijk R, Lounis N, Meyvisch P, Andries K, McNeeley DF 2012 Randomized pilot trial of eight weeks of bedaquiline (TMC207) treatment for multidrug-resistant tuberculosis: long-term outcome, tolerability, and effect on emergence of drug resistance. Antimicrob Agents Chemother 56:3271–3276. doi:10.1128/AAC.06126-11. 22391540 PMC3370813

[14] Guillemont J, Meyer C, Poncelet A, Bourdrez X, Andries K 2011 Diarylquinolines, synthesis pathways and quantitative structure-activity relationship studies leading to the discovery of TMC207. Future Med Chem 3:1345–1360. doi:10.4155/fmc.11.79. 21879841

[15] Food and Drug Administration. 2012 Anti-infective drugs advisory committee meeting briefing document TMC207 (bedaquiline). Treatment of patients with MDR-TB. NDA 204-384 November 28 FDA, Washington, DC.

[16] Tong AST, Choi PJ, Blaser A, Sutherland HS, Tsang SKY, Guillemont J, Motte M, Cooper CB, Andries K, Van den Broeck W, Franzblau SG, Upton AM, Denny WA, Palmer BD, Conole D 2017 6-Cyano analogues of bedaquiline as less lipophilic and potentially safer diarylquinolines for tuberculosis. ACS Med Chem Lett 8:1019–1024. doi:10.1021/acsmedchemlett.7b00196. 29057044 PMC5642017

[17] Choi PJ, Sutherland HS, Tong AST, Blaser A, Franzblau SG, Cooper CB, Lotlikar MU, Upton AM, Guillemont J, Motte M, Queguiner L, Andries K, Van den Broeck W, Denny WA, Palmer BD 2017 Synthesis and evaluation of analogues of the tuberculosis drug bedaquiline containing heterocyclic B-ring units. Bioorg Med Chem Lett 27:5190–5196. doi:10.1016/j.bmcl.2017.10.042. 29107541 PMC5696560

[18] Sutherland HS, Tong AST, Choi PJ, Conole D, Blaser A, Franzblau SG, Cooper CB, Upton AM, Lotlikar MU, Denny WA, Palmer BD 2018 Structure-activity relationships for analogs of the tuberculosis drug bedaquiline with the naphthalene unit replaced by bicyclic heterocycles. Bioorg Med Chem 26:1797–1809. doi:10.1016/j.bmc.2018.02.026. 29482950 PMC5933462

[19] Blaser A, Sutherland HS, Tong AST, Choi PJ, Conole D, Franzblau SG, Cooper CB, Upton AM, Lotlikar M, Denny WA, Palmer BD 2019 Structure-activity relationships for unit C pyridyl analogues of the tuberculosis drug bedaquiline. Bioorg Med Chem 27:1283–1291. doi:10.1016/j.bmc.2019.02.025. 30792104 PMC6467542

[20] Sutherland HS, Tong AST, Choi PJ, Blaser A, Conole D, Franzblau SG, Lotlikar MU, Cooper CB, Upton AM, Denny WA, Palmer BD 2019 3,5-Dialkoxypyridine analogues of bedaquiline are potent antituberculosis agents with minimal inhibition of the hERG channel. Bioorg Med Chem 27:1292–1307. doi:10.1016/j.bmc.2019.02.026. 30803745 PMC6467547

[21] Hotra A, Suter M, Biuković G, Ragunathan P, Kundu S, Dick T, Grüber G 2016 Deletion of a unique loop in the mycobacterial F-ATP synthase gamma subunit sheds light on its inhibitory role in ATP hydrolysis-driven H(+) pumping. FEBS J 283:1947–1961. doi:10.1111/febs.13715. 26996828

[22] Abrams A, Smith JB, Baron C 1972 Carbodiimide-resistant membrane adenosine triphosphatase in mutants of Streptococcus faecalis. I. Studies of the mechanism of resistance. J Biol Chem 247:1484–1488. 4258940

[23] Cohen NS, Lee SH, Brodie AF 1978 Purification and characteristics of hydrophobic membrane protein(s) required for DCCD sensitivity of ATPase in Mycobacterium phlei. J Supramol Struct 8:111–117. doi:10.1002/jss.400080109. 153436

[24] Fillingame RH 1975 Identification of the dicyclohexylcarbodiimide-reactive protein component of the adenosine 5′-triphosphate energy-transducing system of Escherichia coli. J Bacteriol 124:870–883. 126994 10.1128/jb.124.2.870-883.1975 PMC235979

[25] Yoshida M, Poser JW, Allison WS, Esch FS 1981 Identification of an essential glutamic acid residue in the beta subunit of the adenosine triphosphatase from the thermophilic bacterium PS3. J Biol Chem 256:148–153. 6450197

[26] Yoshida M, Allison W, Esch F, Futai M 1982 The specificity of carboxyl group modification during the inactivation of the Escherichia coli F1-ATPase with dicyclohexyl [14C] carbodiimide. J Biol Chem 257:10033–10037. 6213615

[27] Parsonage D, Wilke-Mounts S, Senior AE 1988 E. coli F1‐ATPase: site‐directed mutagenesis of the β‐subunit. FEBS Lett 232:111–114. doi:10.1016/0014-5793(88)80397-1. 2896602

[28] Wu ML, Gengenbacher M, Dick T 2016 Mild nutrient starvation triggers the development of a small-cell survival morphotype in mycobacteria. Front Microbiol 7:947. doi:10.3389/fmicb.2016.00947. 27379076 PMC4909757

[29] Kaser M, Ruf MT, Hauser J, Marsollier L, Pluschke G 2009 Optimized method for preparation of DNA from pathogenic and environmental mycobacteria. Appl Environ Microbiol 75:414–418. doi:10.1128/AEM.01358-08. 19047396 PMC2620729

[30] Delaglio F, Grzesiek S, Vuister GW, Zhu G, Pfeifer J, Bax A 1995 NMRPipe: a multidimensional spectral processing system based on UNIX pipes. J Biomol NMR 6:277–293. doi:10.1007/BF00197809. 8520220

[31] Goddard T, Kneller D 2007 SPARKY. Version 3. University of California, San Francisco, CA.

[32] Moreira W, Aziz DB, Dick T 2016 Boromycin kills mycobacterial persisters without detectable resistance. Front Microbiol 7:199. doi:10.3389/fmicb.2016.00199. 26941723 PMC4761863

