# Supplementary material for: TBAJ-876 Retains Bedaquiline’s Activity against Subunits c and ε of Mycobacterium tuberculosis F-ATP Synthase
Source: Antimicrob Agents Chemother. 2019 Sep 23;63(10):e01191-19. doi: 10.1128/AAC.01191-19 (PMC6761534; doi:10.1128/AAC.01191-19)
Supplement: Supplemental file 1 [file AAC.01191-19-s0001.pdf]

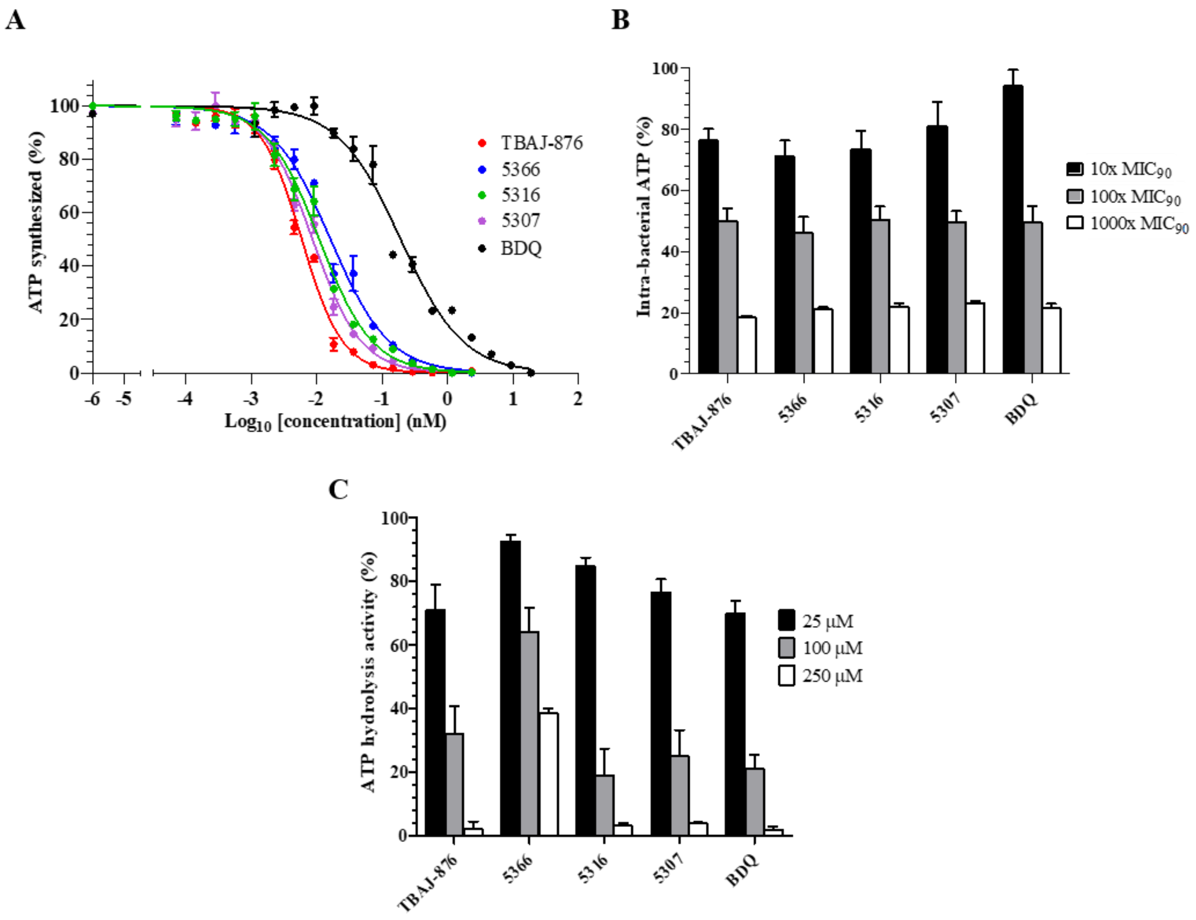

FIG S1 Effect of TBAJ-876 and its analogues on ATP synthesis and hydrolysis in *M. smegmatis*. Effect of the compounds on (A) ATP synthesized by *M. smegmatis* inverted membrane vesicles, (B) intra-bacterial ATP content of whole-cell *M. smegmatis* and (C) ATP hydrolysis activity by *M. smegmatis* inverted membrane vesicles. BDQ was used as a positive control. In (A), the IC_{50} values are as follows: 5.9 pM for TBAJ-876, 16.4 pM for 5366, 11.0 pM for 5316, 8.7 pM for 5307 and 200 pM for BDQ. In (B), the MIC_{90} values are as follows: 6.3 nM for TBAJ-876, 5.5 nM for 5366, 4.6 nM for 5316, 3.5 nM for 5307 and 100 nM for BDQ. In (A – C), all values are represented as a percentage of the drug-free sample which corresponds to 6.29 nmol/mg of protein for (A), 16.2 pmol/ml of culture for (B) and 37.34 µmol/min/mg of protein for (C). For (B), the CFU of all samples were constant at the end of treatment compared to the start of the experiment. Experiments were carried out thrice independently. Results are shown as mean values with standard deviations.

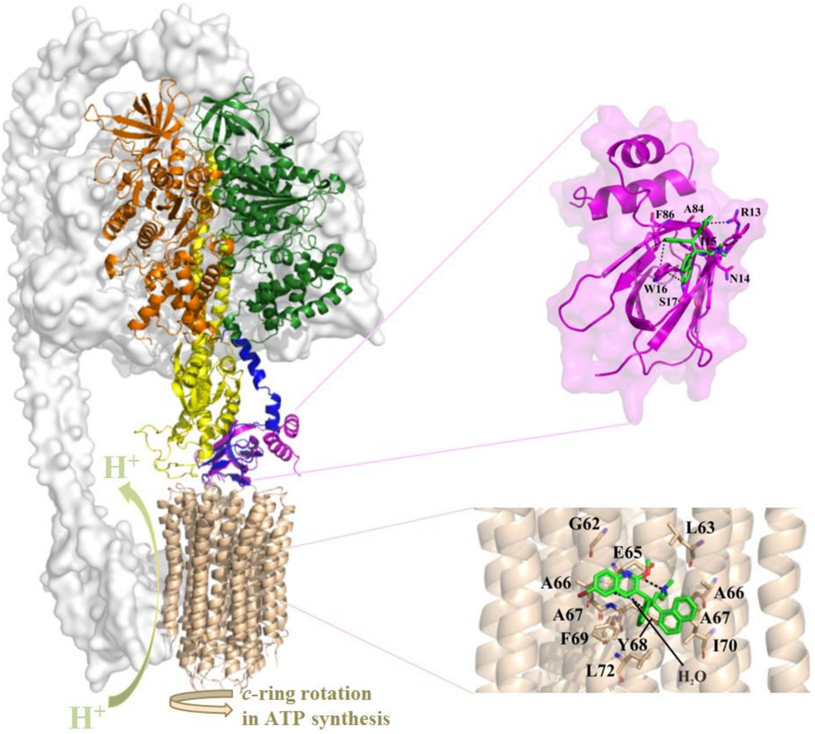

FIG S2 A structural model of the mycobacterial F-ATP synthase and its interaction with BDQ. One α-subunit (*dark green*), one β- subunit (*orange*) and subunits γ (*yellow*) and c (*wheat*) are represented by their respective cartoon representations. The ε-subunit's extended (*blue*) and compact (*magenta*) conformations are also represented by their respective cartoon representations. The subunits *a*, *b*, *b'*, δ and the other α and β subunits are shown as structural surface representations. The interaction profile of BDQ with the *M. phlei* c-ring, based on studies conducted by Preiss et al. (1), is shown in the bottom inset. Two adjacent subunits of the *c*-ring are involved in extensive hydrophobic and hydrophilic interactions with BDQ during drug binding. The residues G62, E65, A66, A67, Y68, F69 and L72 from one subunit and L63, A66, A67 and I70 from another subunit are involved in these interactions. Residue E65, whose counterpart in Mtb is the E61 residue, is involved in hydrogen bond interactions with the dimethylamino moiety and hydroxyl group of BDQ. The interaction profile of BDQ with the Mtb ε-subunit, based on studies conducted by Joon et al. (2), is shown in the top inset. The multitude of aromatic, hydrophobic and hydrogen bonding interactions stabilizing the binding of BDQ with the ε-subunit are presented. Amino acid residues R13, N14 and I15 have hydrogen

bond interactions with the quinoline moiety, dimethylamino group and hydroxyl group of BDQ respectively. Residue W16 has face-to-face stacked interaction with BDQ's naphthalene moiety and phenyl group. Residue F86 has an edge-to-face aromatic interaction with the phenyl group while residues A84 and S17 have hydrophobic interactions with the quinoline and naphthalene moieties of BDQ, respectively.

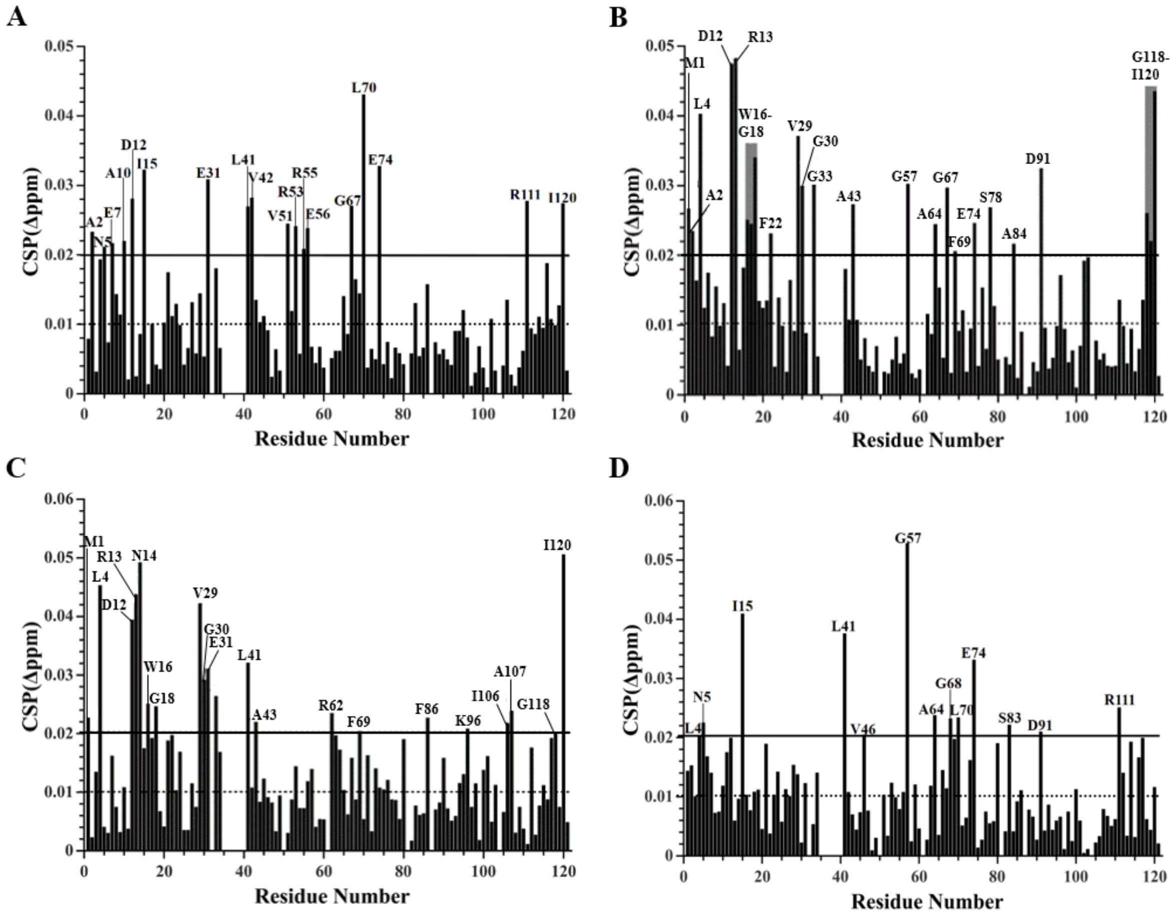

FIG S3 Weighted CSPs for the ^{15}N and ^{1}H resonances of the Mtb ε-subunit in the presence of TBAJ-876 (A), 5366 (B), 5316 (C) and 5307 (D). The dotted line at CSP = 0.01 ppm indicates the average CSP while amino acid residues showing CSP above average + standard deviation (above the solid line at 0.02 ppm) are labelled by their respective one-letter code. The weighted CSPs between the Mtb ε-subunit and compound-bound Mtb ε-subunit, for the backbone ^{15}N and ^{1}H, were calculated by the formula $\Delta\delta = [(\Delta N)^2+(\Delta HN/5)^2]^{0.5}$.

TABLE S1 Primers used in the identification of mutations in the *atpC* and *atpE* genes in the Mtb spontaneous mutants isolated against TBAJ-876 and its analogues

Primer name	Sequence (5' → 3')	Purpose
ATPoperon Frwd	AGGAACCGGTCGCAACTTA	PCR amplification of entire ATP synthase operon
ATPoperon Rev	CGATAACTCAGTGCGAGAACG	PCR amplification of entire ATP synthase operon
atpC Frwd	AAGGGCGATTTCGATCACGTAC	Sequencing of the *atpC* gene
atpC Rev	AGGACAACGACGAGCACGA	Sequencing of the *atpC* gene
atpE Frwd	ACTAGTACCGGATGCTGGTAAC	Sequencing of the *atpE* gene
atpE Rev	AACGTGCCGTTGGGAATGA	Sequencing of the *atpE* gene

REFERENCES

1. Preiss L, Langer JD, Yildiz O, Eckhardt-Strelau L, Guillemont JE, Koul A, Meier T. 2015. Structure of the mycobacterial ATP synthase Fo rotor ring in complex with the anti-TB drug bedaquiline. Sci Adv 1:e1500106.

2. Joon S, Ragunathan P, Sundararaman L, Nartey W, Kundu S, Manimekalai MSS, Bogdanović N, Dick T, Grüber G. 2018. The NMR solution structure of Mycobacterium tuberculosis F-ATP synthase subunit epsilon provides new insight into energy coupling inside the rotary engine. FEBS J 285:1111-1128.
